# Multimodality OCT, IVUS and FFR evaluation of coronary intermediate grade lesions in women vs. men

**DOI:** 10.3389/fcvm.2023.1021023

**Published:** 2023-06-22

**Authors:** Piotr Baruś, Adam Piasecki, Karolina Gumiężna, Adrian Bednarek, Piotr Dunaj, Marcin Głód, Karol Sadowski, Dorota Ochijewicz, Adam Rdzanek, Arkadiusz Pietrasik, Marcin Grabowski, Janusz Kochman, Mariusz Tomaniak

**Affiliations:** First Department of Cardiology, Medical University of Warsaw, Warsaw, Poland

**Keywords:** coronary plaque, sex differences, OCT, FFR, IVUS, stable coronary artery disease

## Abstract

**Background:**

The pathophysiology of atherosclerotic plaque formation and its vulnerability seem to differ between genders due to contrasting risk profiles and sex hormones, however this process is still insufficiently understood. The aim of the study was to compare the differences between sexes regarding the optical coherence tomography (OCT), intravascular ultrasound (IVUS) and fractional flow reserve (FFR)-derived coronary plaque indices.

**Methods:**

In this single-center multimodality imaging study patients with intermediate grade coronary stenoses identified in coronary angiogram (CAG) were evaluated using OCT, IVUS and FFR. Stenoses were considered significant when the FFR value was ≤0.8. Minimal lumen area (MLA), was analyzed by OCT in addition to plaque stratification into fibrotic, calcific, lipidic and thin-cap fibroatheroma (TCFA). IVUS was used for evaluation of lumen-, plaque- and vessel volume, as well as plaque burden.

**Results:**

A total of 112 patients (88 men and 24 women) with chronic coronary syndromes (CCS), who underwent CAG were enrolled. No significant differences in baseline characteristics were present between the study groups. The mean FFR was 0.76 (0.73–0.86) in women and 0.78 ± 0.12 in men (*p *= 0.695). OCT evaluation showed a higher prevalence of calcific plaques among women than men *p *= 0.002 whereas lipid plaques were more frequent in men (*p *= 0.04). No significant differences regarding minimal lumen diameter and minimal lumen area were found between the sexes. In IVUS analysis women presented with significantly smaller vessel area, plaque area, plaque volume, vessel volume (11.1 ± 3.3 mm^2^ vs. 15.0 ± 4.6 mm^2^ *p *= 0.001, 6.04 ± 1.7 mm^2^ vs. 9.24 ± 2.89 mm^2^ *p *< 0.001, 59.8 ± 35.2 mm^3^ vs. 96.3 (52.5–159.1) mm^3^ *p *= 0.005, 106.9 ± 59.8 mm^3^ vs. 153.3 (103–253.4) mm^3^ *p *= 0.015 respectively). At MLA site plaque burden was significantly greater for men than women (61.50 ± 7.7% vs. 55.5 ± 8.0% *p *= 0.005). Survival did not differ significantly between women and men (94.6 ± 41.9 months and 103.51 ± 36.7 months respectively; *p *= 0.187).

**Conclusion:**

The presented study did not demonstrate significant differences in FFR values between women and men, yet a higher prevalence of calcific plaques by OCT and lower plaque burden at the MLA site by IVUS was found in women vs. men.

## 1. Introduction

The pathophysiology of atherosclerotic plaque formation and its vulnerability seem to be different between genders due to contrasting risk profiles and sex hormones (1–3). However, this process is still insufficiently understood. There is still a limited amount of data on sex associated differences in plaque morphology and their influence on blood flow dynamics, underscoring the need for further research.

The primary modality for diagnosing coronary artery disease (CAD) is coronary angiogram (CAG) (4–6). However, it has several widely acknowledged limitations (4–6). It is estimated that even 50% of patients who suffered from cardiac arrest did not experience any premonitory symptoms (7). Therefore, additional techniques have been developed in order to deepen the diagnostic process and optimize treatment strategy, i.e., intravascular ultrasound (IVUS), optical coherence tomography (OCT) and fractional flow reserve (FFR).

IVUS has a tissue penetration depth of up to 6 mm, enabling a full-thickness visualization of the vessel wall (8). Nevertheless, its resolution remains relatively low (axial 100–150 µm and lateral 150–300 µm, at 40 MHz) (9), as compared to infrared light-based OCT providing very high resolution (axial 10–20 µm and lateral 20–90 µm), though with a penetration depth of 1–2 mm (10–18).

Therefore, direct visualization of the artery wall is feasible, enabling a precise evaluation of plaque composition and its superficial layers (e.g., thin cap fibroatheroma – TCFA, plaque rapture) (19–21). Moreover, intravascular modalities are recognized to positively impact the clinical outcomes regarding CAD assessment and PCI guidance (10, 22). On the other hand, FFR/iFR remain the guideline-recommended invasive modalities to identify coronary lesions requiring interventional procedures to resolve myocardial ischemia (23, 24).

The aim of the study was to visualize and compare the imaging (OCT, IVUS) and functional indices of coronary lesions in women vs. men, taking into account such parameters as minimal lumen area (MLA), plaque characteristics (fibrotic, calcific, lipidic or TCFA), plaque burden and a functional index of FFR among patients undergoing CAG due to chronic coronary syndromes (CCS).

## 2. Material and methods

### 2.1. Study population

**Figure 1:**
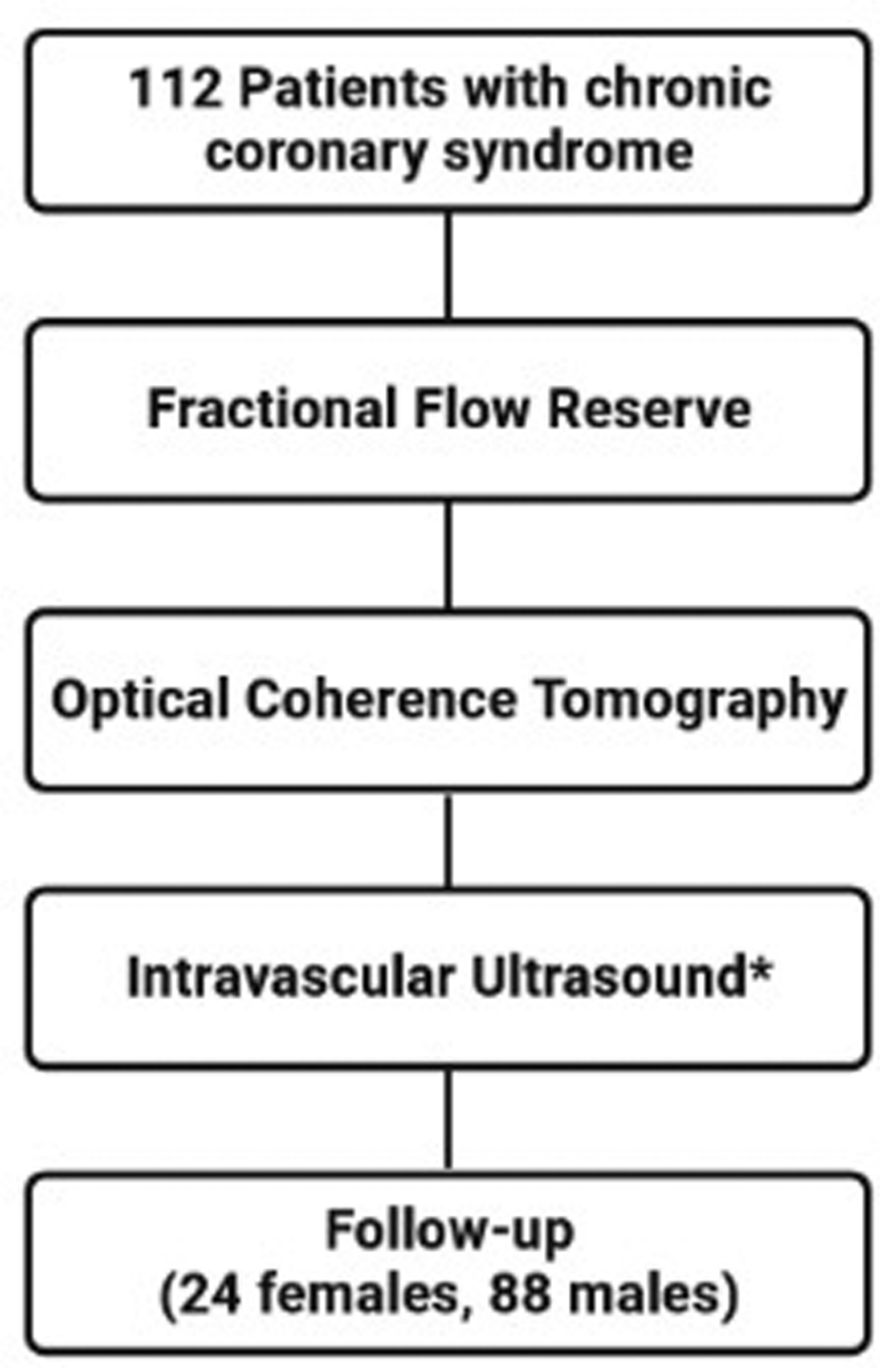
Study flow chart. Intravascular ultrasound was performed at the discretion of the operator (in 64 patients).

This was a single-center, prospective, observational, longitudinal, cohort study that enrolled patients with CCS (*n* = 112) who underwent CAG. Intermediate grade coronary stenoses were evaluated with FFR, OCT and IVUS (Figure 1). The relevance of the stenoses was found significant if FFR ≤ 0.8. The study protocol was approved by the local Ethics Committee.

The study inclusion and exclusion criteria have been previously published (25). In brief, the inclusion criteria comprised: chronic coronary syndrome, presence of chest pain ranked 2-3 in the Canadian Cardiovascular Society classification or positive ischemia test (exercise test, single photon emission tomography – SPECT), age >18 years, intermediate grade coronary stenoses of 40%–80% evaluated visually during angiography (26), FFR and OCT examination of the same lesion.

Exclusion criteria: left main disease, ostial right coronary lesion, bypass graft lesions, hemodynamic instability, acute or chronic renal insufficiency defined as serum creatinine level >1.5 mmol/L, contraindication for adenosine administration, pregnancy.

### 2.2. OCT

OCT recordings were obtained with a commercially available frequency domain OCT imaging system (Abbott, C7XR Dragonfly TM, LightLab Imaging Inc., MA, USA), using the non-occlusive technique (4, 10, 16, 22).

**Figure 2:**
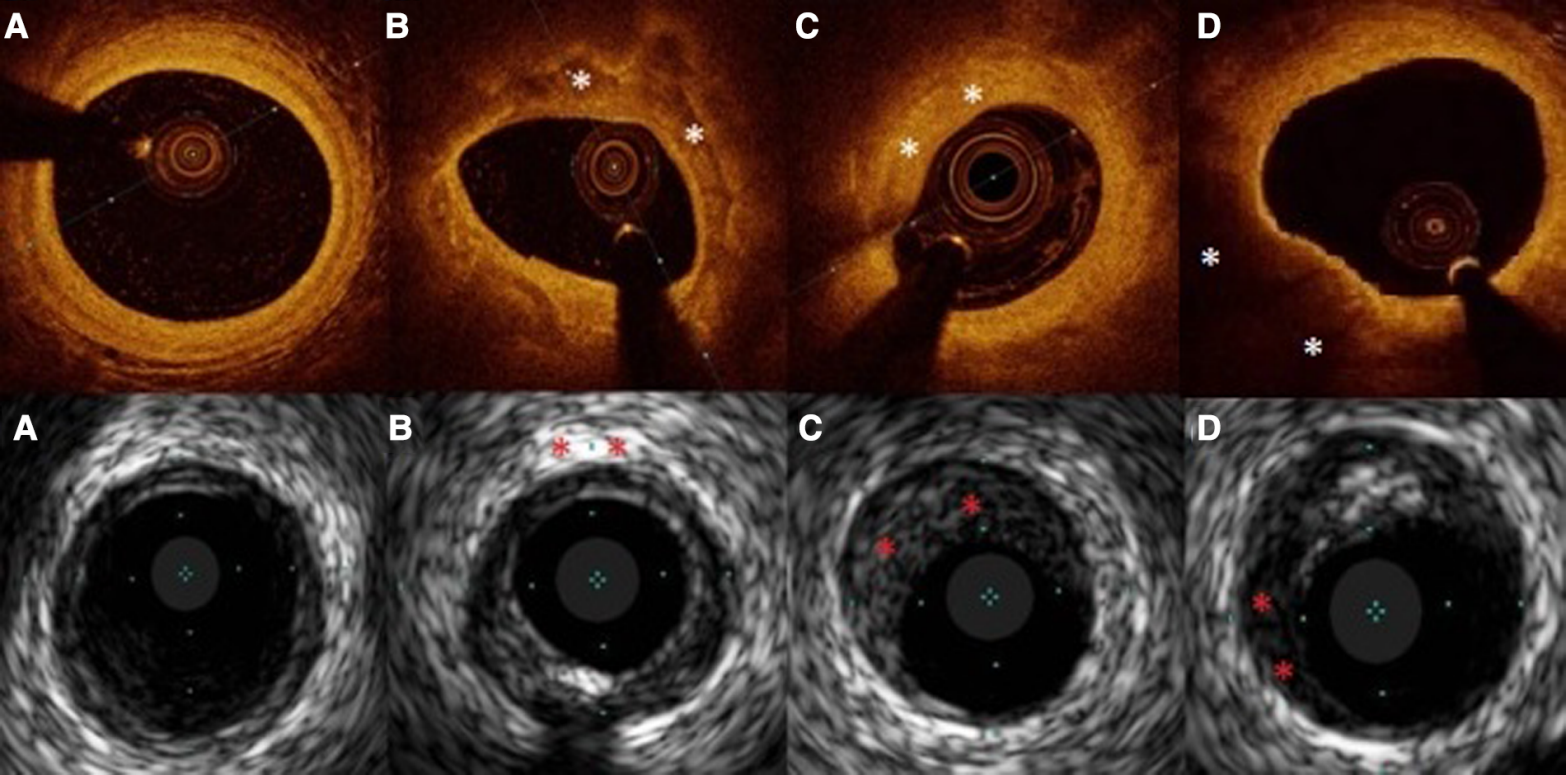
Examples of OCT and IVUS obtained images. In capital letters (**A–D**) OCT images, in small letters (**a–d**) IVUS images. Plaques marked with “*”. (**A**), (a)-healthy vessel, (**B**) calcific plaque, (**b**)- calcification, (**C**) fibrous plaque, (**c**)- fibrous plaque, (**D**) lipidic plaque, (**d**)-lipidic area in plaque.

OCT images were analyzed according to expert consensuses’ definitions (27–30), by the analysts blinded to patient characteristics, IVUS and FFR result. Evaluation of the reference lumen area was performed in the largest lumen proximal or distal to a stenosis (within 10 mm of the stenosis). Morphometric assessment of the plaque was done at the site of MLA in at least three consecutive frames. Plaques were stratified into fibrous, calcified, lipid-rich or mixed. Fibrous plaque is characterized by high backscattering and a relatively homogenous signal, calcified plaque comprises calcium visible as a signal poor heterogeneous region with sharply delineated border (27–30) (Figure 2). In addition, the calcium angle (the circumference of the calcium covering the lumen and presented in degrees) was assessed. Plaque was considered lipid-rich in case of inhomogeneous signal-poor region with diffused borders (28, 31). The lipid angle was computed as the arc of a low-signal region presented in degrees. Fibrous cap thickness (FCT) was defined as the distance between the arterial lumen and the inner border of the lipid or calcium pool. The FCT was assessed first at 0.2-mm intervals over the plaque and then 3 times at its thinnest part at each cross-section, and the average value was taken into the final analyses (31). TCFA was defined with minimal FCT < 65 µm.

### 2.3. FFR

**Figure 3:**
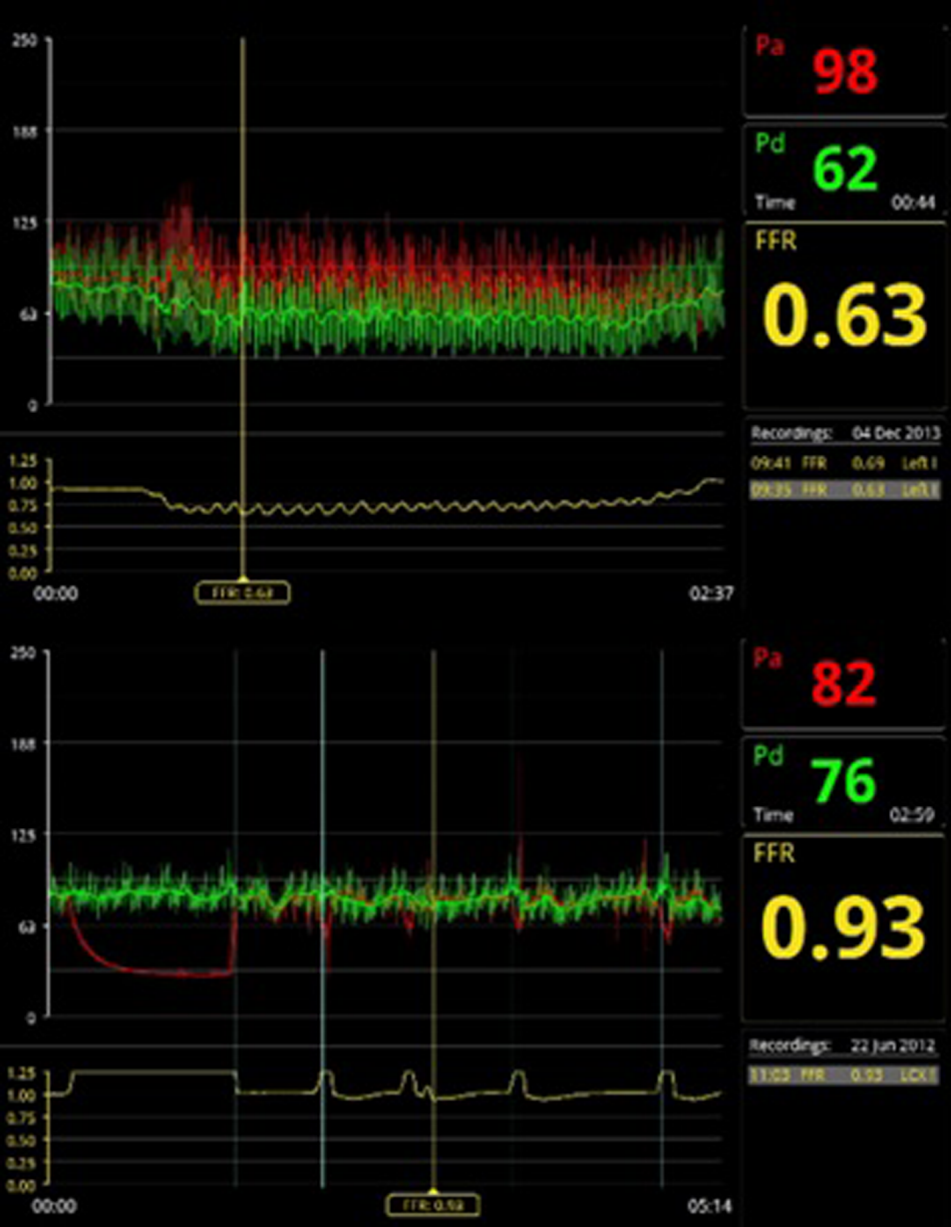
Examples of FFR measurements. On the top panel a significant FFR value of 0.63, on the bottom panel a non-significant FFR value of 0.93.

Coronary pressure was obtained using a 0.014-inch pressure guide wire (St. Jude Medical, Minneapolis, MN, USA). Maximal hyperemia was induced by intravenous adenosine administration at 140 µg/kg/min through a large peripheral vein. The used formula for FFR calculations was mean hyperemic distal coronary pressure divided by mean aortic pressure. The stenosis was found significant in the case of a FFR ≤ 0.80 (32–34) (Figure 3).

### 2.4. IVUS

In order to acquire an IVUS image, the catheter was placed in the distal fragment of the vessel and a pullback was performed at a speed of 0.5 mm/s at 40 MHz (35). IVUS image assessment was performed in 0.5 mm intervals using a dedicated software by analyst blinded to patients characteristics, FFR and OCT results. The plaque burden at MLA site was calculated using the formula: (external elastic membrane area-lumen area)/external elastic membrane area × 100%.

### 2.5. Statistical analysis

Statistical analyses have been performed using the SPSS version 28.0 (IBM Corp, Armonk, NY, USA). The distribution was analyzed with the Kolmogorov–Smirnov test. Normally distributed data were presented with means and standard deviation (SD), whereas the non-parametric data were presented with median and percentiles 25th and 75th (interquartile range). Categorical variables were presented by percentages within each group. Between-group comparisons were carried out with a Student *t*-test or Mann-Whitney U test for continuous variables and Chi-square test or Fisher's exact test, as appropriate, for categorical variables. Kaplan-Meier analysis with log-rank test was conducted to compare the MACE-free survival and death probability between the sexes. The results were considered significant for *p* < 0.05.

## 3. Results

### 3.1. Patients characteristics

A total of 112 patients (132 lesions) that underwent CAG were included in this study. This group comprised 24 women (21.4%) and 88 men (78.6%). The prevalence of coexisting conditions was high with the two most common, hypertension and hypercholesterolemia, present in 84.0% and 66.0% of patients respectively. There were no significant differences between genders in terms of age and comorbidities in the analyzed cohort. No differences in prior medication use were recorded either (Table 1).

**Table 1:** Baseline patient characteristics.

Characteristic	Women (*n *= 24)	Men (*n *= 88)	*p*-value
Age – mean years	65.17 ± 9.9	64.74 ± 9.6	0.425
Coexisting conditions – no. (%)			
Atrial fibrillation	4 (16.7)	13 (14.8)	0.758
Hypertension	22 (91.7)	72 (81.8)	0.353
Hypercholesterolemia	18 (75.0)	56 (63.6)	0.297
Diabetes mellitus	9 (37.5)	27 (30.7)	0.526
Chronic Kidney Disease	1 (4.2)	11 (12.5)	0.456
Peripheral Artery Disease	3 (12.5)	8 (9.1)	0.700
Heart failure	1 (4.2)	7 (8.0)	1.000
Previous stroke or transient ischemic attack	1 (4.2)	2 (2.3)	0.519
Previous myocardial infarction	15 (62.5)	46 (52.3)	0.373
Active smokers – no. (%)	3 (12.5)	16 (18.2)	0.760
Previous intervention – no. (%)			
Percutaneous Coronary Intervention	19 (79.2)	63 (71.6)	0.458
Coronary Artery Bypass Grafting	0	4 (4.5)	0.576
Medications – no. (%)			
β-blockers	23 (95.8)	84 (95.5)	1.000
Calcium channel blockers	8 (33.3)	23 (26.1)	0.485
ACE-inhibitors	17 (70.8)	59 (67.0)	0.725
ARB	6 (25.0)	17 (19.3)	0.573
Aspirin	22 (91.7)	87 (98.9)	0.115
Clopidogrel	16 (66.7)	55 (62.5)	0.784
Statin	23 (95.8)	85 (96.6)	1.000
NOAC	0	1 (1.1)	1.000
VKA	2 (8.3)	11 (12.5)	0.731

ARB, angiotensin II receptor blocker; NOAC, non-vitamin K antagonist oral anticoagulants; VKA, vitamin K antagonists.

There were no differences in plaque localization between men and women. Most frequently lesions were located in left anterior descending (LAD), which was the case for 20 women and 58 men. The mean FFR was 0.76 (0.73–0.86) for women and 0.78 ± 0.12 for men and did not differ significantly (Table 2).

**Table 2:** Angiographic findings.

Plaque location – no. (%)	Women (*n *= 27)	Men (*n *= 105)	*p*-value
Left main stem	0 (0.0)	5 (4.8)	0.583
Left anterior descending	20 (74.1)	58 (55.2)	0.076
Circumflex	2 (7.4)	13 (12.4)	0.735
Marginal branch	1 (3.7)	5 (4.8)	1.000
Right coronary artery	4 (14.8)	24 (22.9)	0.362
FFR	0.76 (0.73–0.86)	0.78 ± 0.12	0.695

FFR, fractional flow reserve.

### 3.2. IVUS

In a subset of 64 patients (16 women and 48 men) parameters were assessed using IVUS (Table 3). Women had a significantly smaller vessel area and plaque area. There were no significant differences in lumen volume, but plaque volume and vessel volume were significantly greater in men. MLA did not differ between genders. At the MLA site, plaque burden was significantly greater for men than women. The same was true for vessel area, plaque area and average intimal thickness at MLA site.

**Table 3:** Lesion characteristic by IVUS.

Variables	Women (*n *= 16)	Men (*n *= 48)	*p*-value
Lumen volume mm^3^	47.1 ± 26.4	56.2 (32.8–97.9)	0.097
Plaque volume mm^3^	59.8 ± 35.2	96.3 (52.5–159.1)	0.005
Vessel volume mm^3^	106.9 ± 59.8	153.3 (103.0–253.4)	0.015
Lumen area mm^2^	5.02 ± 2.16	5.17 (4.09–6.90)	0.198
Vessel area mm^2^	11.1 ± 3.3	15 ± 4.6	0.001
Plaque area mm^2^	6.04 ± 1.7	9.24 ± 2.89	<0.001
Plaque burden %	55.5 ± 8	61.5 ± 7.7	0.005
At minimal lumen area			
Minimal lumen area mm^2^	2.76 (2.1–4.71)	3.12 (2.39–3.96)	0.768
Vessel area mm^2^	10.04 ± 3.3	14.22 ± 5.02	0.001
Plaque area mm^2^	6.42 ± 2.08	10.67 ± 4.2	<0.001
Plaque burden %	65.03 ± 10.12	74.0 ± 9.26	<0.001
Average intimal thickness mm	0.70 ± 0.18	0.99 ± 0.29	<0.001

The values are provided as mean ± SD or median (IQR).

### 3.3. OCT

Plaques were classified into calcified, fibrous, mixed and lipidic. The most common type overall was calcified plaque, which was observed in 49 lesions – 17 in women and 32 in men. This plaque type was more common in women than men. Lipidic plaques on the other hand were more prevalent in men than women, but the type most frequently found in men was fibrous type – 37 plaques accounting for 35.9% of plaques (Table 4).

**Table 4:** Plaque categories by OCT.

Plaque type – no. (%)	Women (*n *= 27)	Men (*n *= 103)	*p*-value
Calcified	17 (63.0)	32 (31.1)	0.002
Fibrous	8 (29.6)	37 (35.9)	0.541
Mixed	2 (7.4)	19 (18.4)	0.242
Lipidic	0 (0.0)	15 (14.6)	0.04

The lesion characteristics by OCT are presented in Table 5. The length of the lesion did not differ significantly between men and women. Minimal lumen diameter and minimal lumen area did not show any significant differences as well. There were no significant differences in mean, minimal or maximal angle of calcium.

**Table 5:** Lesion characteristic by OCT.

Variable	Women (*n *= 27)	Men (*n *= 105)	*p*-value
Length mm	15.57 ± 8.3	11.6 (6.4–17.65)	0.089
Minimal lumen diameter mm	1.39 (0.92–1.48)	1.30 (1.1–1.58)	0.299
Mean lumen area mm^2^	3.25 ± 1.23	3.62 (2.81–4.86)	0.035
Minimal lumen area mm^2^	1.82 ± 0.83	1.88 (1.46–2.66)	0.131
Mean cap thickness over calcium mm	0.081 ± 0.048	0.12 ± 0.076	0.006
Mean angle of calcium	119 ± 56	95 (69–131)	0.277
Maximal angle of calcium	128 ± 67	96.8 (73.5–172)	0.681
Minimal angle of calcium	112 ± 50	81 (60–112)	0.07
Thrombus no.	3	11	1.000
Cholesterol crystals no.	3	17	0.736
Macrophages no.	3	13	1.000
Presence of TCFA no.	4	19	0.783

### 3.4. Follow-up

The clinical follow-up was present for 94 patients (median follow up 122 months, IQR = 107–122 months). Out of them, 25 patients died (26.6%) – 7 women and 18 men, no difference in the overall mortality was found for women vs. men (31.8% vs. 25%, *p* = 0.526). Average survival (in months) did not differ significantly between women and men (94.6 ± 41.9 and 103.51 ± 36.7 respectively; *p *= 0.187). Similarly, there were no significant differences in the number of major adverse cardiac event (MACE), defined as: all-cause death, myocardial infarction, repeated revascularization, stroke and hospitalization due to heart failure (Supplementary Table S1).

**Figure 4:**
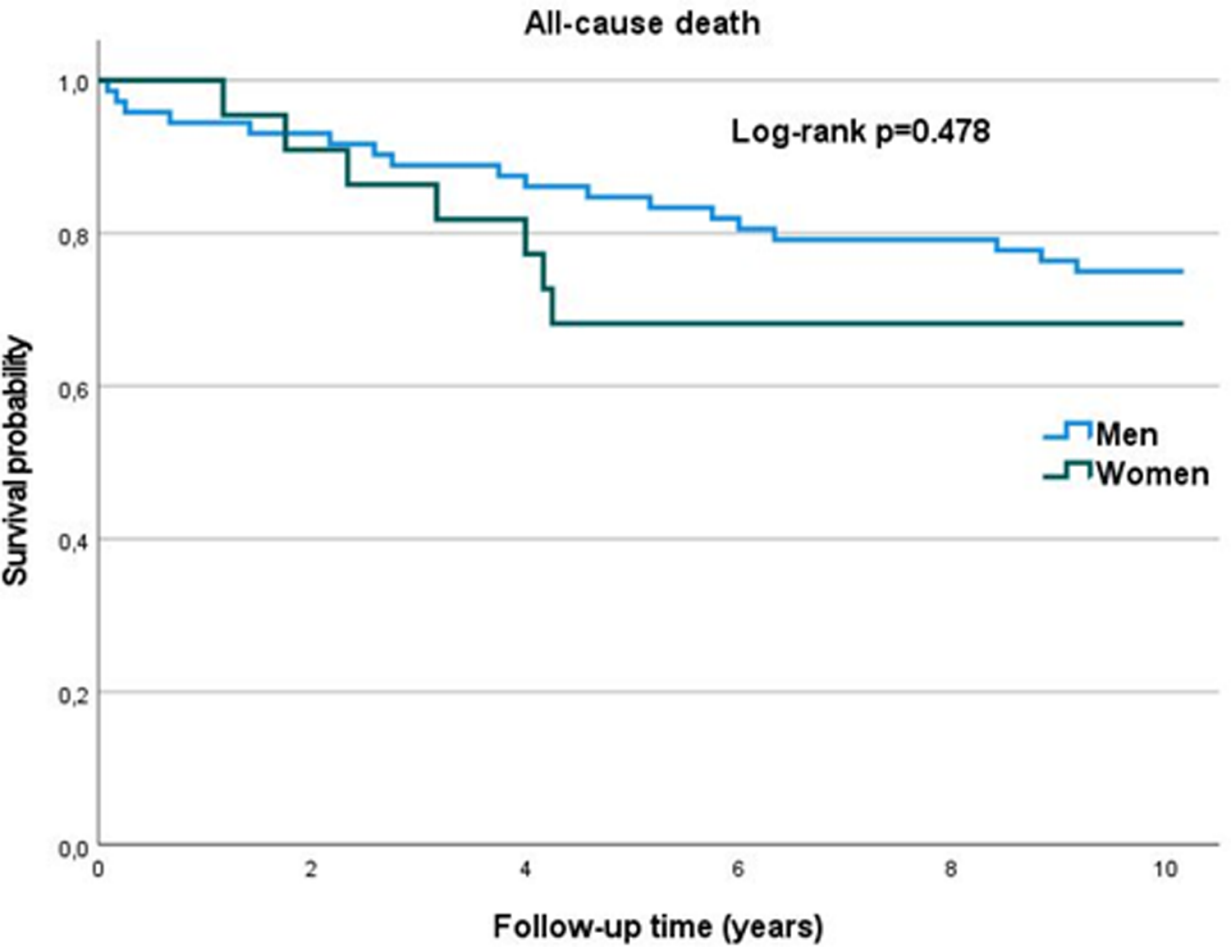
Kaplan-Meier curve – overall survival (years).

There were no significant differences in the mortality rates and survival probability (Figure 4) between men and women. MACE occurrence also did not differ significantly between sexes.

## 4. Discussion

Enriching a standard CAG with intravascular imaging modalities such as OCT or IVUS allows a more thorough lesion analysis and an identification of factors that worsen patients’ prognosis e.g., TCFA (20, 21), macrophage infiltration (36) or lipid rich plaques (37).

The results obtained using OCT show a statistically significant difference in the most common type of plaque between sexes. In our study 63% of enrolled women had a calcified plaque, whereas among men such a plaque was present in 31.1% of the cases (*p *= 0.002). Furthermore, lipidic plaque was more common in men (*p* = 0.04).

The prevalence of each plaque type in women vs. men varies between the hitherto studies. A study by Mariani et al. analyzing coronary arteries by OCT in patients with stable CAD (138 men and 42 women) showed a higher percentage of lipid rich plaques and macrophages in women (38). On the other hand, in a study by Giordana et al., women presenting with non-ST segment elevation myocardial infarction (NSTEMI) had a lower prevalence of lipid plaques (39). Similar conclusions were drawn from a study that analyzed 187 non-culprit lesions among patients with CAD, women had overall a lower lipid index and less lipid-core length (40), which is consistent with our findings. In our study the mean age for women and men is 65.17 and 64.74 years respectively. A study by Sato et al. showed, that in the case of a group of patients below the age of 70 years calcifications are present more often among women (41), what is consistent with our results. A study by Kataoka et al. evaluated differences between genders in stable CAD and acute coronary syndromes (ACS) regarding OCT indices. A total number of 320 and 115 lesions in CCS and ACS respectively, were taken into consideration. In women presenting with CAD and ACS a lower prevalence of cholesterol crystals and calcifications was observed (42). The results of the above-mentioned studies and our findings are often contrary, thus proving the need for a more thorough assessment on a larger patients sample in order to develop a more gender specific approach in CAD treatment resulting in better clinical outcomes.

Previous studies concerning gender differences in IVUS plaque morphology have reached similar conclusions. Vessel area has been repeatedly found to be smaller in women than men (43, 44). A study by Kang et al. assessed vessel area and plaque burden at minimum lumen area (MLA) site with both measurements greater in men (44). The same study as well as Bharadwaj et al. study found that plaque burden was greater in men in reference vessel area (44, 45). The results in our study are consistent with the above. Vessel area and plaque area overall were found to be greater in men than women. At minimal lumen area vessel area, plaque area and plaque burden were also significantly greater in men than women. Moreover, this study has found the average intimal thickness at MLA to be greater in men than women, which was not reported in any previous study concerning this issue.

The main finding of our study concerning FFR was that on average the FFR in patients undergoing CAG due to CAD does not differ significantly by gender. FFR-guided PCI is superior to classical coronary angiography. It allows to identify the hemodynamically significant lesions and therefore, to choose a more adequate treatment strategy (46–48). It was already established, that it is equally beneficial for both male and female patients with CAD, although the FFR below 0.8 has a higher positive prognostic value in men (49). Moreover, the FFR values below 0.8 are associated with a higher risk of death or MI in female patients (50).

The studies to date suggest that with the same grade of stenosis in coronary arteries, women tend to have higher values of FFR (44, 51), which seems to be associated with the size of the body and hence the mass of the heart, and not as it was previously suspected with microvascular dysfunction (44, 52, 53). The composition of the plaque may also impact FFR value. Non-calcified and low-density plaques are associated with lower FFRs in both patients with and without hemodynamically significant narrowing of the vessel (54). A recent study showed that after adjustment for left ventricle mass and plaque characteristics, sex is not an independent factor of FFR value (53). In our population, there were no significant differences in baseline characteristics between genders, including the factors associated with the progression of CAD such as age, HT, or dyslipidemia, which is different in comparison with previously conducted studies. This would stay in line with the conclusions that gender is not an independent factor of FFR values and the higher FFR values in women (49) are due to other differences in populations’ characteristics. Even though the plaque composition differed significantly in terms of calcification, the FFR remained on the same levels between sex groups which may suggest that the calcification grade did not significantly impact the outcomes.

It should be noted that our findings have limitations. We conducted a single-center and non-randomized study; thus, it is imperative that further research include more centers and randomization. Moreover, there is a high disproportion between genders as women comprise only 21.4% of the investigated group, thus future research should focus on minimizing that disparity.

Performing a vessel analysis by both OCT and IVUS requires two separate catheters. It is associated with difficulties in an accurate lesion analysis due to an imperfection of image overlapping and carries a higher risk of side effects (55, 56). However, both modalities are complementary with each other, therefore combined catheters were designed, which perform an evaluation of the same area at the same time, enabling a complete vessel wall visualization and precise evaluation (19, 56). Furthermore, during the last few years new diagnostic techniques have been developed in order to assess coronary blood flow disturbances and estimate FFR without using a pressure wire. Such modalities build a three-dimensional artery model based on CAG images. Recent studies proved similar sensitivity and specificity of these techniques in comparison to pressure wire-based FFR (57–61).

## 5. Conclusions

The presented study did not demonstrate significant differences in FFR values between women and men, yet a higher prevalence of calcific plaques by OCT and lower plaque burden at the MLA site by IVUS was found in women as compared to men.

## Data Availability

The original contributions presented in the study are included in the article/Supplementary Material, further inquiries can be directed to the corresponding author.

## References

[1] Mendelsohn ME Karas RH. Molecular and cellular basis of cardiovascular gender differences. Science. (2005) 308(5728):1583–7. 10.1126/science.1112062 15947175

[2] Caboral MF. Update on cardiovascular disease prevention in women. Am J Nurs. (2013) 113(3):26–33; quiz, 44. 10.1097/01.NAJ.0000427876.02924.dd 23411578

[3] Bavry AA Limacher MC. Prevention of cardiovascular disease in women. Semin Reprod Med. (2014) 32(6):447–53. 10.1055/s-0034-1384628 25321422

[4] Collet JP Thiele H Barbato E Barthelemy O Bauersachs J Bhatt DL 2020 ESC guidelines for the management of acute coronary syndromes in patients presenting without persistent ST-segment elevation. Eur Heart J. (2021) 42(14):1289–367. 10.1093/eurheartj/ehaa575 32860058

[5] Ibanez B James S Agewall S Antunes MJ Bucciarelli-Ducci C Bueno H 2017 ESC guidelines for the management of acute myocardial infarction in patients presenting with ST-segment elevation. Rev Esp Cardiol (Engl Ed). (2017) 70(12):1082. 10.1016/j.recesp.2017.10.048 29198432

[6] Knuuti J Wijns W Saraste A Capodanno D Barbato E Funck-Brentano C 2019 ESC guidelines for the diagnosis and management of chronic coronary syndromes. Eur Heart J. (2020) 41(3):407–77. 10.1093/eurheartj/ehz425 31504439

[7] Tomaniak M Katagiri Y Modolo R de Silva R Khamis RY Bourantas CV Vulnerable plaques and patients: state-of-the-art. Eur Heart J. (2020) 41(31):2997–3004. 10.1093/eurheartj/ehaa227 32402086 PMC8453282

[8] Maehara A Matsumura M Ali ZA Mintz GS Stone GW. IVUS-guided versus OCT-guided coronary stent implantation: a critical appraisal. JACC Cardiovasc Imaging. (2017) 10(12):1487–503. 10.1016/j.jcmg.2017.09.008 29216976

[9] Sung JH Jeong JS. Development of high-frequency (>60 MHz) intravascular ultrasound (IVUS) transducer by using asymmetric electrodes for improved beam profile. Sensors (Basel). (2018) 18(12):4414. 10.3390/s18124414 30551639 PMC6308511

[10] Ali ZA Karimi Galougahi K Mintz GS Maehara A Shlofmitz RA Mattesini A. Intracoronary optical coherence tomography: state of the art and future directions. EuroIntervention. (2021) 17(2):e105–23. 10.4244/EIJ-D-21-00089 34110288 PMC9725016

[11] Neumann FJ Sousa-Uva M Ahlsson A Alfonso F Banning AP Benedetto U 2018 ESC/EACTS guidelines on myocardial revascularization. Eur Heart J. (2019) 40(2):87–165. 10.1093/eurheartj/ehy394 30165437

[12] Ali ZA Maehara A Genereux P Shlofmitz RA Fabbiocchi F Nazif TM Optical coherence tomography compared with intravascular ultrasound and with angiography to guide coronary stent implantation (ILUMIEN III: OPTIMIZE PCI): a randomised controlled trial. Lancet. (2016) 388(10060):2618–28. 10.1016/S0140-6736(16)31922-5 27806900

[13] Kim N Lee JH Jang SY Bae MH Yang DH Park HS Intravascular modality-guided versus angiography-guided percutaneous coronary intervention in acute myocardial infarction. Catheter Cardiovasc Interv. (2020) 95(4):696–703. 10.1002/ccd.28359 31132217

[14] Maehara A Ben-Yehuda O Ali Z Wijns W Bezerra HG Shite J Comparison of stent expansion guided by optical coherence tomography versus intravascular ultrasound: the ILUMIEN II study (observational study of optical coherence tomography [OCT] in patients undergoing fractional flow reserve [FFR] and percutaneous coronary intervention). JACC Cardiovasc Interv. (2015) 8(13):1704–14. 10.1016/j.jcin.2015.07.024 26585621

[15] van Zandvoort LJC Ali Z Kern M van Mieghem NM Mintz GS Daemen J Improving PCI outcomes using postprocedural physiology and intravascular imaging. JACC Cardiovasc Interv. (2021) 14(22):2415–30. 10.1016/j.jcin.2021.08.069 34794649

[16] Pawlowski T Legutko J Kochman J Roleder T Pregowski J Chmielak Z Clinical use of intracoronary imaging modalities in Poland. Expert opinion of the association of cardiovascular interventions of the polish cardiac society. Kardiol Pol. (2022) 80(4):509–19. 10.33963/KP.a2022.0071 35290660

[17] Johnson TW Raber L di Mario C Bourantas C Jia H Mattesini A Clinical use of intracoronary imaging. Part 2: acute coronary syndromes, ambiguous coronary angiography findings, and guiding interventional decision-making: an expert consensus document of the European association of percutaneous cardiovascular interventions. Eur Heart J. (2019) 40(31):2566–84. 10.1093/eurheartj/ehz332 31112213

[18] Baruś P Modrzewski J Gumiężna K Dunaj P Głód M Bednarek A Comparative appraisal of intravascular ultrasound and optical coherence tomography in invasive coronary imaging: 2022 update. J Clin Med. (2022) 11(14):4055. 10.3390/jcm11144055 35887819 PMC9324054

[19] Ono M Kawashima H Hara H Gao C Wang R Kogame N Advances in IVUS/OCT and future clinical perspective of novel hybrid catheter system in coronary imaging. Front Cardiovasc Med. (2020) 7:119. 10.3389/fcvm.2020.00119 32850981 PMC7411139

[20] Fabris E Berta B Roleder T Hermanides RS AJJ IJ Kauer F Thin-cap fibroatheroma rather than any lipid plaques increases the risk of cardiovascular events in diabetic patients: insights from the COMBINE OCT-FFR trial. Circ Cardiovasc Interv. (2022) 15(5):e011728. 10.1161/CIRCINTERVENTIONS.121.011728 35485232

[21] Kedhi E Berta B Roleder T Hermanides RS Fabris E AJJ IJ Thin-cap fibroatheroma predicts clinical events in diabetic patients with normal fractional flow reserve: the COMBINE OCT-FFR trial. Eur Heart J. (2021) 42(45):4671–9. 10.1093/eurheartj/ehab433 34345911

[22] Oosterveer TTM van der Meer SM Scherptong RWC Jukema JW. Optical coherence tomography: current applications for the assessment of coronary artery disease and guidance of percutaneous coronary interventions. Cardiol Ther. (2020) 9(2):307–21. 10.1007/s40119-020-00185-4 32564339 PMC7584694

[23] Peper J Becker LM van Kuijk JP Leiner T Swaans MJ. Fractional flow reserve: patient selection and perspectives. Vasc Health Risk Manag. (2021) 17:817–31. 10.2147/VHRM.S286916 34934324 PMC8684425

[24] Achenbach S Rudolph T Rieber J Eggebrecht H Richardt G Schmitz T Performing and interpreting fractional flow reserve measurements in clinical practice: an expert consensus document. Interv Cardiol. (2017) 12(2):97–109. 10.15420/icr.2017:13:2 29588737 PMC5808579

[25] Tomaniak M Ochijewicz D Koltowski L Rdzanek A Pietrasik A Jakala J OCT-derived plaque morphology and FFR-determined hemodynamic relevance in intermediate coronary stenoses. J Clin Med. (2021) 10(11):2379. 10.3390/jcm10112379 34071299 PMC8197966

[26] Kennedy MW Fabris E Ijsselmuiden AJ Nef H Reith S Escaned J Combined optical coherence tomography morphologic and fractional flow reserve hemodynamic assessment of non- culprit lesions to better predict adverse event outcomes in diabetes mellitus patients: COMBINE (OCT-FFR) prospective study. Rationale and design. Cardiovasc Diabetol. (2016) 15(1):144. 10.1186/s12933-016-0464-8 27724869 PMC5057218

[27] Prati F Guagliumi G Mintz GS Costa M Regar E Akasaka T Expert review document part 2: methodology, terminology and clinical applications of optical coherence tomography for the assessment of interventional procedures. Eur Heart J. (2012) 33(20):2513–20. 10.1093/eurheartj/ehs095 22653335 PMC3470836

[28] Prati F Regar E Mintz GS Arbustini E Di Mario C Jang IK Expert review document on methodology, terminology, and clinical applications of optical coherence tomography: physical principles, methodology of image acquisition, and clinical application for assessment of coronary arteries and atherosclerosis. Eur Heart J. (2010) 31(4):401–15. 10.1093/eurheartj/ehp433 19892716

[29] Raber L Mintz GS Koskinas KC Johnson TW Holm NR Onuma Y Clinical use of intracoronary imaging. Part 1: guidance and optimization of coronary interventions. An expert consensus document of the European association of percutaneous cardiovascular interventions. Eur Heart J. (2018) 39(35):3281–300. 10.1093/eurheartj/ehy285 29790954

[30] Tearney GJ Regar E Akasaka T Adriaenssens T Barlis P Bezerra HG Consensus standards for acquisition, measurement, and reporting of intravascular optical coherence tomography studies: a report from the international working group for intravascular optical coherence tomography standardization and validation. J Am Coll Cardiol. (2012) 59(12):1058–72. 10.1016/j.jacc.2011.09.079 22421299

[31] Kini AS Vengrenyuk Y Yoshimura T Matsumura M Pena J Baber U Fibrous cap thickness by optical coherence tomography in vivo. J Am Coll Cardiol. (2017) 69(6):644–57. 10.1016/j.jacc.2016.10.028 27989887

[32] Götberg M Christiansen EH Gudmundsdottir IJ Sandhall L Danielewicz M Jakobsen L Instantaneous wave-free ratio versus fractional flow reserve to guide PCI. [1533−4406 (Electronic)]. 10.1056/NEJMoa1616540 28317438

[33] Pijls NH Tanaka N Fearon WF. Functional assessment of coronary stenoses: can we live without it? Eur Heart J. (2013) 34(18):1335–44. 10.1093/eurheartj/ehs436 23257950

[34] Sels JW Tonino PA Siebert U Fearon WF Van't Veer M De Bruyne B Fractional flow reserve in unstable angina and non-ST-segment elevation myocardial infarction experience from the FAME (fractional flow reserve versus angiography for multivessel evaluation) study. JACC Cardiovasc Interv. (2011) 4(11):1183–9. 10.1016/j.jcin.2011.08.008 22115657

[35] Balocco S Gatta C Ciompi F Wahle A Radeva P Carlier S Standardized evaluation methodology and reference database for evaluating IVUS image segmentation. Comput Med Imaging Graph. (2014) 38(2):70–90. 10.1016/j.compmedimag.2013.07.001 24012215

[36] Gatto L Alfonso F Paoletti G Burzotta F La Manna A Budassi S Relationship betweeen the amount and location of macrophages and clinical outcome: subanalysis of the CLIMA-study. Int J Cardiol. (2022) 346:8–12. 10.1016/j.ijcard.2021.11.042 34798205

[37] Xing L Higuma T Wang Z Aguirre AD Mizuno K Takano M Clinical significance of lipid-rich plaque detected by optical coherence tomography: a 4-year follow-up study. J Am Coll Cardiol. (2017) 69(20):2502–13. 10.1016/j.jacc.2017.03.556 28521888

[38] Mariani L Burzotta F Aurigemma C Romano A Niccoli G Leone AM Frequency-domain optical coherence tomography plaque morphology in stable coronary artery disease: sex differences. Coron Artery Dis. (2017) 28(6):472–7. 10.1097/MCA.0000000000000522 28644210

[39] Giordana F Errigo D D'Ascenzo F Montefusco A Garbo R Omede P Female sex impact on culprit plaque at optical coherence tomography analysis in the setting of acute coronary syndrome in OCT-FORMIDABLE registry. Future Cardiol. (2020) 16(2):123–31. 10.2217/fca-2018-0073 31965820

[40] Tian J Wang X Tian J Yu B. Gender differences in plaque characteristics of nonculprit lesions in patients with coronary artery disease. BMC Cardiovasc Disord. (2019) 19(1):45. 10.1186/s12872-019-1023-5 30808307 PMC6390304

[41] Sato T Minami Y Asakura K Katamine M Kato A Katsura A Age- and gender-related differences in coronary lesion plaque composition on optical coherence tomography. Circ J. (2020) 84(3):463–70. 10.1253/circj.CJ-19-0859 31983726

[42] Kataoka Y Puri R Hammadah M Duggal B Uno K Kapadia SR Sex differences in nonculprit coronary plaque microstructures on frequency-domain optical coherence tomography in acute coronary syndromes and stable coronary artery disease. Circ Cardiovasc Imaging. (2016) 9(8):e004506. 10.1161/CIRCIMAGING.116.004506 27511975

[43] Nakamura T Ogita M Ako J Momomura S. Gender differences of plaque characteristics in elderly patients with stable angina pectoris: an intravascular ultrasonic radiofrequency data analysis. Int J Vasc Med. (2010) 2010:134692. 10.1155/2010/134692 21253514 PMC3022163

[44] Kang SJ Ahn JM Han S Lee JY Kim WJ Park DW Sex differences in the visual-functional mismatch between coronary angiography or intravascular ultrasound versus fractional flow reserve. JACC Cardiovasc Interv. (2013) 6(6):562–8. 10.1016/j.jcin.2013.02.016 23787231

[45] Bharadwaj AS Vengrenyuk Y Yoshimura T Baber U Hasan C Narula J Multimodality intravascular imaging to evaluate sex differences in plaque morphology in stable CAD. JACC Cardiovasc Imaging. (2016) 9(4):400–7. 10.1016/j.jcmg.2016.02.007 27052268

[46] Tonino PA De Bruyne B Pijls NH Siebert U Ikeno F van' t Veer M Fractional flow reserve versus angiography for guiding percutaneous coronary intervention. N Engl J Med. (2009) 360(3):213–24. 10.1056/NEJMoa0807611 19144937

[47] Curzen N Rana O Nicholas Z Golledge P Zaman A Oldroyd K Does routine pressure wire assessment influence management strategy at coronary angiography for diagnosis of chest pain?: the RIPCORD study. Circ Cardiovasc Interv. (2014) 7(2):248–55. 10.1161/CIRCINTERVENTIONS.113.000978 24642999

[48] Van Belle E Rioufol G Pouillot C Cuisset T Bougrini K Teiger E Outcome impact of coronary revascularization strategy reclassification with fractional flow reserve at time of diagnostic angiography: insights from a large French multicenter fractional flow reserve registry. Circulation. (2014) 129(2):173–85. 10.1161/CIRCULATIONAHA.113.006646 24255062

[49] Kim HS Tonino PA De Bruyne B Yong AS Tremmel JA Pijls NH The impact of sex differences on fractional flow reserve-guided percutaneous coronary intervention: a FAME (fractional flow reserve versus angiography for multivessel evaluation) substudy. JACC Cardiovasc Interv. (2012) 5(10):1037–42. 10.1016/j.jcin.2012.06.016 23078733

[50] Li J Rihal CS Matsuo Y Elrashidi MY Flammer AJ Lee MS Sex-related differences in fractional flow reserve-guided treatment. Circ Cardiovasc Interv. (2013) 6(6):662–70. 10.1161/CIRCINTERVENTIONS.113.000762 24149976 PMC3904351

[51] Fairbairn TA Dobson R Hurwitz-Koweek L Matsuo H Norgaard BL Ronnow Sand NP Sex differences in coronary computed tomography angiography-derived fractional flow reserve: lessons from ADVANCE. JACC Cardiovasc Imaging. (2020) 13(12):2576–87. 10.1016/j.jcmg.2020.07.008 32861656

[52] Crystal GJ Klein LW. Fractional flow reserve: physiological basis, advantages and limitations, and potential gender differences. Curr Cardiol Rev. (2015) 11(3):209–19. 10.2174/1573403X10666141020113318 25329922 PMC4558352

[53] Kim CH S Y Zhang J Differences in plaque characteristics and myocardial mass: implications for physiological significance. JACC Asia. (2022) 2(2):157–67. 10.1016/j.jacasi.2021.11.011 36339124 PMC9627886

[54] Gaur S Ovrehus KA Dey D Leipsic J Botker HE Jensen JM Coronary plaque quantification and fractional flow reserve by coronary computed tomography angiography identify ischaemia-causing lesions. Eur Heart J. (2016) 37(15):1220–7. 10.1093/eurheartj/ehv690 26763790 PMC4830909

[55] Raber L Heo JH Radu MD Garcia-Garcia HM Stefanini GG Moschovitis A Offline fusion of co-registered intravascular ultrasound and frequency domain optical coherence tomography images for the analysis of human atherosclerotic plaques. EuroIntervention. (2012) 8(1):98–108. 10.4244/EIJV8I1A16 22580254

[56] Zeng Y Tateishi H Cavalcante R Tenekecioglu E Suwannasom P Sotomi Y Serial assessment of tissue precursors and progression of coronary calcification analyzed by fusion of IVUS and OCT: 5-year follow-up of scaffolded and nonscaffolded arteries. JACC Cardiovasc Imaging. (2017) 10(10 Pt A):1151–61. 10.1016/j.jcmg.2016.11.016 28330651

[57] Neleman T Scoccia A Masdjedi K Tomaniak M Ligthart JMR Witberg KT The prognostic value of angiography-based vessel fractional flow reserve after percutaneous coronary intervention: the FAST outcome study. Int J Cardiol. (2022) 359:14–9. 10.1016/j.ijcard.2022.04.021 35421516

[58] Scoccia A Tomaniak M Neleman T Groenland FTW Plantes A Daemen J. Angiography-based fractional flow reserve: state of the art. Curr Cardiol Rep. (2022) 24(6):667–78. 10.1007/s11886-022-01687-4 35435570 PMC9188492

[59] Neleman T Masdjedi K Van Zandvoort LJC Tomaniak M Ligthart JMR Witberg KT Extended validation of novel 3D quantitative coronary angiography-based software to calculate vFFR: the FAST EXTEND study. JACC Cardiovasc Imaging. (2021) 14(2):504–6. 10.1016/j.jcmg.2020.08.006 33011122

[60] Rubimbura V Guillon B Fournier S Amabile N Chi Pan C Combaret N Quantitative flow ratio virtual stenting and post stenting correlations to post stenting fractional flow reserve measurements from the DOCTORS (Does Optical Coherence Tomography Optimize Results of Stenting) study population. [1522-726X (Electronic)]. 10.1002/ccd.28615 31763775

[61] Tomaniak M Neleman T Ziedses des Plantes A Masdjedi K van Zandvoort LJC Kochman J Diagnostic accuracy of coronary angiography-based vessel fractional flow reserve (vFFR) Virtual stenting. LID – 10.3390/jcm11051397 [doi] LID – 1397. [2077-0383 (Print)]. 10.3390/jcm11051397 PMC8910880 35268488

