# Supplementary material for: Multimodality OCT, IVUS and FFR evaluation of coronary intermediate grade lesions in women vs. men
Source: Front Cardiovasc Med. 2023 Jun 22;10:1021023. doi: 10.3389/fcvm.2023.1021023 (PMC10325624; doi:10.3389/fcvm.2023.1021023)
Supplement: Supplementary file 1 [file Table1.docx]

|  | Women (*n*=22) | Men (*n*=72) | *p*-value |
| --- | --- | --- | --- |
| Death | 7 | 18 | 0.526 |
| Average survival time months | 94.6±41.9 | 103.51±36.7 | 0.187 |
| MACE | 11 | 32 | 0.314 |
| Average time to MACE months | 47.3±30.9 | 49.8±34.3 | 0.426 |
| MI | 1 | 5 | 1.000 |
| Stroke | 2 | 3 | 0.228 |
| HF hospitalization | 1 | 10 | 0.656 |
| PCI | 2 | 9 | 1.000 |
| CABG | 0 | 3 | 1.000 |

Supplementary table 1. Clinical outcomes (*n* = 94). MACE – major adverse cardiac event (all-cause death, myocardial infarction, repeated revascularization, stroke and hospitalization due to heart failure), MI – myocardial infarction, HF – heart failure, PCI – percutaneous coronary intervention, CABG – coronary artery bypass graft.
